# Circatrigintan instead of lunar periodicity of larval release in a brooding coral species

**DOI:** 10.1038/s41598-018-23274-w

**Published:** 2018-04-04

**Authors:** Bart Linden, Jef Huisman, Baruch Rinkevich

**Affiliations:** 1 Israel Oceanographic and Limnological Research, National Institute of Oceanography, Tel-Shikmona, P.O. Box 8030, Haifa, 31080 Israel; 2 Department of Freshwater and Marine Ecology, Institute for Biodiversity and Ecosystem Dynamics (IBED), University of Amsterdam, P.O. Box 94248, 1090 GE Amsterdam, The Netherlands

## Abstract

Larval release by brooding corals is often assumed to display lunar periodicity. Here, we show that larval release of individual *Stylophora pistillata* colonies does not comply with the assumed tight entrainment by the lunar cycle, and can better be classified as a circatrigintan pattern. The colonies exhibited three distinct reproductive patterns, characterized by short intervals, long intervals and no periodicity between reproductive peaks, respectively. Cross correlation between the lunar cycle and larval release of the periodic colonies revealed an approximately 30-day periodicity with a variable lag of 5 to 10 days after full moon. The observed variability indicates that the lunar cycle does not provide a strict zeitgeber. Other factors such as water temperature and solar radiation did not correlate significantly with the larval release. The circatrigintan patterns displayed by *S. pistillata* supports the plasticity of corals and sheds new light on discussions on the fecundity of brooding coral species.

## Introduction

Biological properties of coral gametes, embryos, planula larvae and recruits are often considered important in interpreting ecological trajectories towards the resilience of coral reefs^1^. Sexual reproduction in reef corals contributes to the genetic variance, population robustness and overall healthy growth of coral reefs^2–4^. Sexual maturation, onset of reproduction, timing of spawning, biological clocks and other intrinsic traits are vital reproductive properties^5–7^. In addition, sexual reproduction in reef corals is constrained by extrinsic factors such as location^8^, food availability^9^, competitive interactions^10–12^, anthropogenic activities^13^, water temperature, solar radiation, tidal pressure^2,7,14–16^, and more. How the intrinsic and extrinsic factors determine the resultant reproductive patterns in coral colonies remains a subject of debate^17^, but being able to accurately predict reproductive patterns of corals can be an invaluable tool for reef management and restoration^2,8,18–23^.

Circalunar periodicity is here defined as periodic behaviour with a ca. 29.5-day frequency^24^ in which the lunar cycle acts as a zeitgeber. Circa 30-day cycles that are not influenced by the lunar phase and irradiance intensity are best characterised as circatrigintan^24^ (ca. 30-day periodicity). Though there is no doubt about the circannual and circadian rhythm of sexual reproduction in most coral species, a convincing demonstration of lunar periodicity^25^ in broadcasting, shedding or spawning of some corals remains elusive^2,7,26,27^. Populations of some brooding corals, such as *Pocillopora damicornis*^8^, have been demonstrated to have a circalunar reproductive pattern (~29.5 days) that is not necessarily correlated to lunar irradiance intensity. This result makes lunar irradiance an unlikely zeitgeber. In some brooding coral species (e.g., *Stylophora pistillata*^27–30^), planulae timing and releases are predicted to happen on a daily basis during certain months, revealing peaks and troughs in their reproductive intensity. These patterns are believed to be associated with environmental factors and constraints that are different from those dictating the germ line release documented in broadcasting species^2,7,30,31^. The determination of an exact spawning event for some broadcasting coral species, such as *Acropora palmata* in the Caribbean, is done with some degree of accuracy^32,33^. This is also true for the synchronized mass spawning phenomenon of a consortium of >140 coral species in the Great Barrier Reef, Australia^34,35^.

The common Indo-Pacific branching coral species *S. pistillata* from the Gulf of Eilat (Aqaba), Red Sea, is a hermaphroditic brooding species with a long reproductive season (December/January to July/September^13,30,36,37^). While long-term shifts in sexual reproduction patterns, such as reproductive seasonality and reproductive efforts, were documented for *S. pistillata* populations from Eilat^37^ in the last three decades, the possible association of lunar periodicity with larval release in this species has been the subject of some debate. Several authors^13,31,36^ could not find conclusive evidence for lunar periodicity in planulation, timing and shed of larval numbers from gravid *S. pistillata* colonies, whereas other studies^30,38^ claimed to reveal such lunar periodicity in larval release. This study investigates this deliberation of circalunar periodicity in *S. pistillata* reproduction as displayed by shallow water colonies from Eilat.

## Results

**Figure 1:**
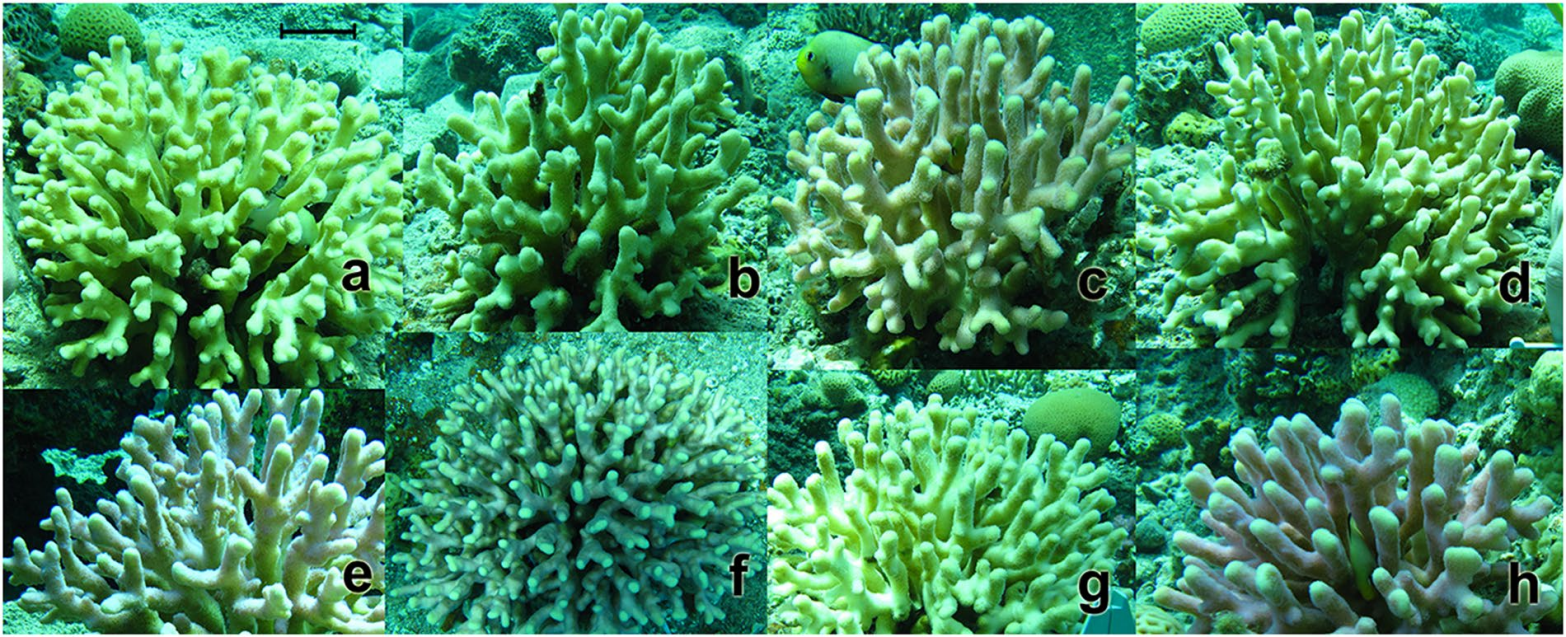
The eight gravid *Stylophora pistillata* colonies chosen for planulae collection. The colonies differed in size and colour, representing the morphological diversity of *S. pistillata* colonies at Eilat. A plastic ruler was used to estimate the diameter of each colony, with (**a**–**h**) representing colonies #1 to #8. The black scale bar in (**a**) indicates a length of 3 cm.

**Figure 2:**
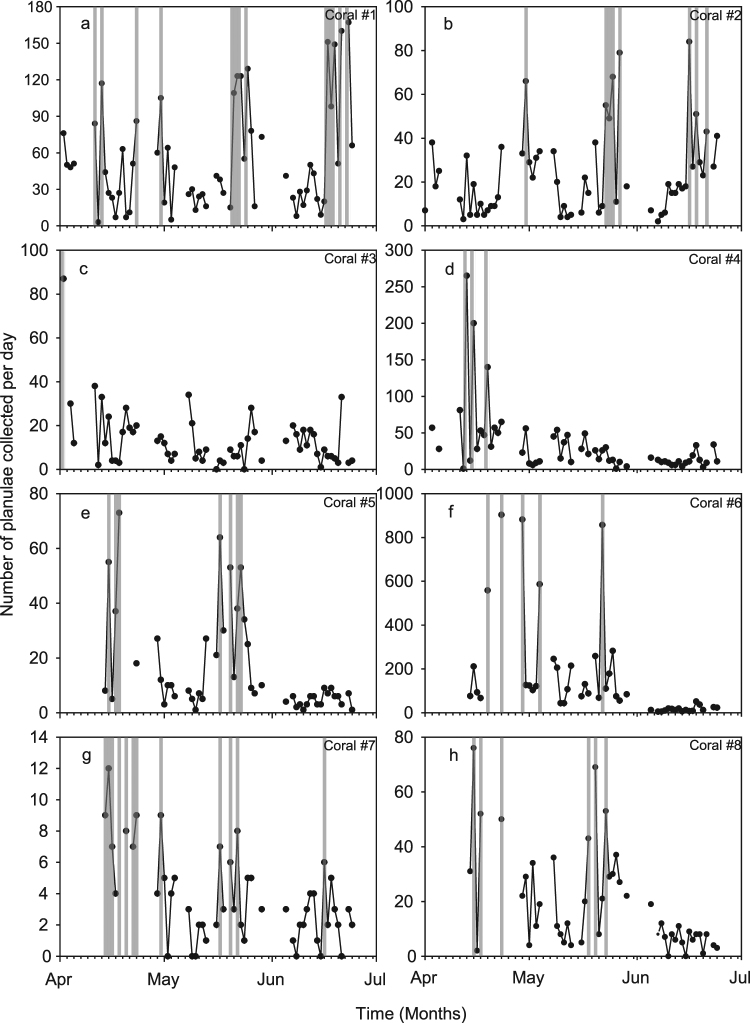
Daily collections of planulae for each of the eight coral colonies (**a**–**h**) of *S. pistillata* during April–July. Peaks are indicated with light grey highlights and are defined by number of planulae collected being greater than or equal to 50% of the maximum number of planulae collected on a single day during the study of that particular colony (see Table 1).

Larvae were collected from eight colonies and counted per colony per day (during 48–59 nights per colony, over a total period of 84 days). In total, 16,586 planulae were collected during 427 sampling sessions (Table 1). The numbers of planulae released per colony per day varied greatly among the eight coral colonies (Fig. 1). Planula numbers varied between maximum peaks of 903 (colony #6) to 12 (colony #7) planulae caught on a single night from a specific colony (Fig. 2). The most gravid colony (#6) released on average 156.1 planulae per day (ranging from 5 to 903 on a collection night), whereas the least productive colony (#7) released on average 3.8 planulae per day (ranging from 0 to 12 on a collection night; Table 1). The average number of planulae collected per colony per day increased with colony volume (Pearson Correlation: r = 0.81; n = 8, p(1-tailed) = 0.007).

**Table 1:** Reproductive effort of eight *S. pistillata* colonies (#1–8; see Fig. 1) during the reproductive season (April–June).

Coral #	Collecting nights	Average planulae/day (±sd)	Colony ecological volume (L)	Planulae total	Maximum planulae/day	Planulae/ecological volume (L)/day
**1**	59	53.7 (±43.3)	6.74	3170	167	7.97
**2**	59	23.2 (±19.3)	6.86	1361	84	3.36
**3**	58	13.6 (±13.6)	7.79	789	87	1.75
**4**	57	33.3 (±45.8)	12.65	1898	265	2.63
**5**	48	15.8 (±18.1)	4.77	759	73	3.31
**6**	48	156.1 (±226.2)	17.28	7493	903	9.03
**7**	50	3.8 (±2.9)	5.60	189	12	0.68
**8**	48	19.3 (±18.3)	2.30	927	76	8.39

Ecological volume as described by Shafir *et al*.^46^.

**Figure 3:**
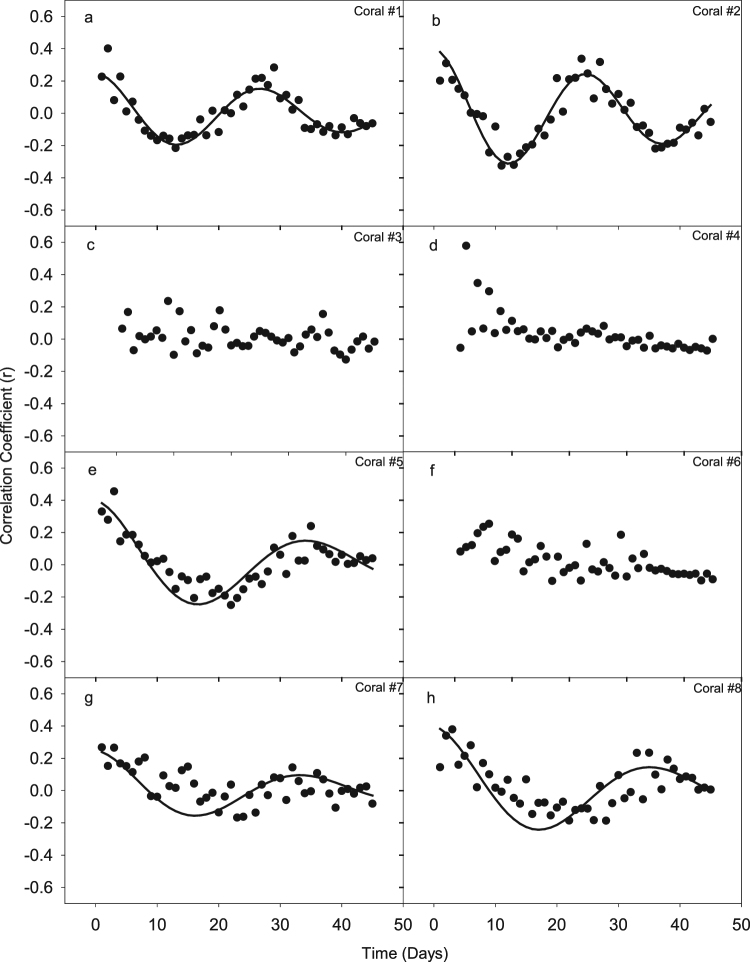
Auto-correlation analyses of planulae released from the eight *S. pistillata* colonies (**a**–**h**). Time lag was set at a maximum of 45 days. Each point in the graph represents the correlation coefficient of the data at time t + n compared to the data at time t, where n is the time lag in days. Coral colonies #1, 2, 5, 7 and 8 show a clear wave pattern, indicative of recurrence in released planulae behaviours. Trend lines were composed of damped sine waves and used to approximate period.

Auto-correlation analysis revealed three distinct patterns of peak larval release (Fig. 3). The first pattern was exhibited by colonies (#1) and (#2), which had a short peak-to-peak period of approximately 27 and 25 days, respectively. The second pattern was exhibited by colonies (#5), (#7) and (#8), which had a long peak-to-peak period of 34, 33 and 35 days respectively. The third pattern was exhibited by colonies (#3), (#4) and (#6), which did not display any repetition of planulae releasing peaks during the April-June reproductive period (Fig. 3).

Coral fecundity was high during the studied reproductive season. In April, 98.2% of the 109 collected samples contained planulae larvae, a trend that was repeated in the following months (97.1% of the 175 samples and 96.5% of the 143 samples for May and June, respectively). Coral colonies #1 and #2 released greater numbers of planulae as the season progressed, whereas the other six *S. pistillata* colonies released lower numbers of planulae as the season progressed (Table 2).

**Table 2:** Average number of planulae released per colony per day ± SD during the reproductive season (April–June) Planulae.

*Coral #*	Average planulae per day		
	April	May	June
**1**	49.5 ± 33.7	49.9 ± 39.5	62.9 ± 56.0
**2**	18.5 ± 16.3	25.8 ± 21.3	24.9 ± 19.9
**3**	21.0 ± 19.5	9.7 ± 8.6	11.0 ± 8.1
**4**	70.2 ± 69.4	21.6 ± 16.0	12.7 ± 8.5
**5**	24.1 ± 24.1	20.0 ± 18.3	4.7 ± 2.5
**6**	351.3 ± 341.6	184.1 ± 192.2	16.5 ± 12.2
**7**	7.2 ± 2.9	3.20 ± 2.3	2.4 ± 1.7
**8**	30.4 ± 24.1	23.1 ± 17.3	6.8 ± 4.6

**Figure 4:**
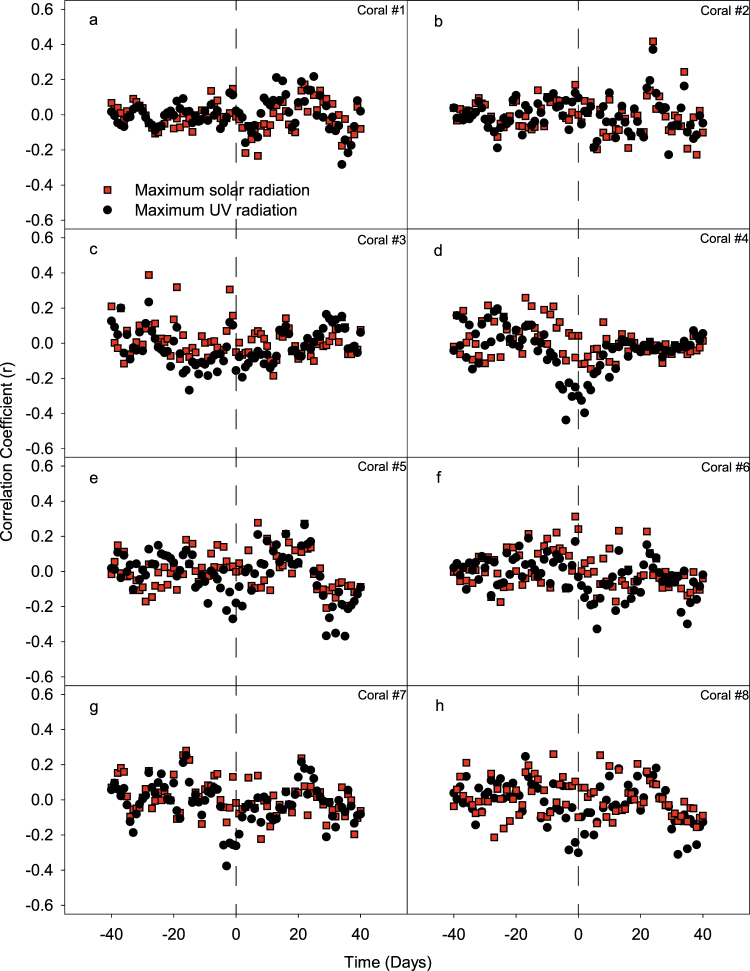
Cross-correlation of larval release versus daily maximum solar irradiance (black triangles) and daily maximum UV irradiance (red circles), for each of the eight coral colonies (**a**–**h**). Time lag was set at a maximum of ±40 days. Each point in the graph represents the correlation coefficient between the number of planulae at time t and the solar (or UV) radiation at time t ± n, where n is the time lag in days. No distinct pattern was found and no period could be established.

**Figure 5:**
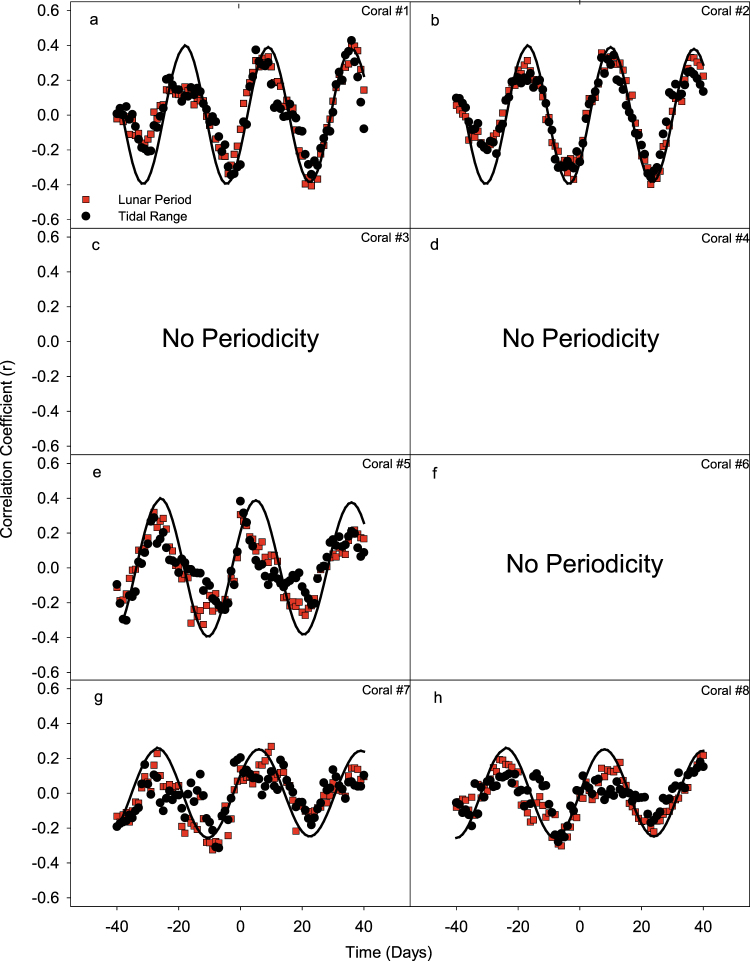
Cross-correlation of larval release versus lunar period (red squares) and tidal range (black circles), for each of the eight coral colonies (**a**–**h**). Time lag was set at a maximum of ±40 days. Each point in the graph represents the correlation coefficient between the number of planulae at time t and the lunar period (or tidal range) at time t ±n, where n is the time lag in days. Trend lines were composed of sine waves and used to approximate the periodicity of coral colonies that had repeating larval spawning peaks. Coral colonies #3, 4 and 6 did not show periodicity (see Fig. 3), and were therefore left out of the cross-correlation analyses.

Cross-correlation analysis revealed no tangible link between *S. pistillata* larval releases and daily maximum solar irradiance or UV irradiance (Fig. 4). Cross-correlation analysis of the 5 periodic colonies showed coherent oscillations of *S. pistillata* larval releases with the lunar period and tidal range, characterized by a variable periodicity of 27 to 33 days and a variable time lag of 5 to 10 days after full moon depending on the colony (Fig. 5).

**Figure 6:**
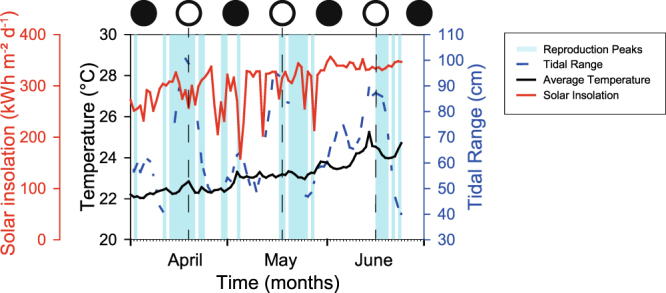
Compilation of reproduction peaks and concomitant changes of tidal range (green line, solid), average water temperature (black line, solid), daily solar insolation (red line, solid) and full moon phases (black line, dashed). The light grey areas comprise the reproductive peaks of all eight *S. pistillata* colonies (see Fig. 2). Full moon occurred on the 18^th^ of April, 17^th^ of May, and 15^th^ of June.

In total, peaks in larval release were not related to water temperature or solar irradiance. They were loosely associated with the lunar cycle, occurred during both neap and spring tide, and both before and after full moon (Fig. 6).

## Discussion

This is the first time, to our knowledge, that the planulae release of individual coral colonies has been monitored*, in situ*, for a prolonged period (84 days). A key advantage of this time series approach is that it enables assessment of possible periodicities in planulae releases for individual brooding corals. All other studies on *S. pistillata* thus far have randomly sampled and subsequently grouped collections of planulae produced by multiple colonies over a prolonged period and reported those results^13,27,30,37^.

Our results show large variation in planula release per colony, which has also been observed in previous studies of *S. pistillata* at the same site. For instance, Rinkevich & Loya^13,39^ reported that planula release per colony per day was 24.2 ± 42.5 (mean ± SD), collected periodically every 3–4 days every month, in the years 1974–75. Furthermore, planula release per colony per day declined from 56.9 ± 20.8 in March 2006 to 18.3 ± 2.9 in July 2006^37^, which is comparable to the seasonal decline from April to June 2011 observed in our study (Table 2). Hence, our results are consistent with previous studies.

Of the eight coral colonies monitored in our study, three had no discernible periodicity and five had consecutive peak larval releases during the sampling period; two of these five coral colonies displayed a period of 25 and 27 days, while the other three coral colonies had periods of 33–35 days. This variation in the timing of planulae release does not support population-wide synchronization of planulae release to a zeitgeber. Despite earlier suggestions that water temperature and UV or solar radiation might be potentially important environmental drivers of larval release periodicity^2,17,31,40,41^, our results did not reveal such a relationship with these variables. It is possible that due to the limitations of field work and the high variability of planulation a local peak in larval release might have been missed during days when no traps were placed in the field.

Cross-correlation analysis showed a relation between the reproductive patterns of the periodic coral colonies and the lunar cycle (~29.5 days) as well as between reproductive patterns of the periodic coral colonies and the spring-neap tidal cycle (as captured by the variation in tidal range). The periodicities of these cross-correlations varied from 27 to 33 days, with a time lag of 5 to 10 days depending on the colony, and none of the coral colonies actually showed a ~29.5-day periodicity in larval release. Consequently, peaks in larval release gradually shifted phase with respect to the lunar cycle, and occurred during both spring tide and neap tide, and both before and after full moon (Fig. 6).

It could be argued that *S. pistillata* in the Gulf of Eilat (Aqaba) is loosely circalunar, having approximately 29.5-day peak larval release cycles when averaged over all periodic colonies (Fig. 5), but the large lunar phase variation (from well before full moon to new moon and spring to neap tide; Fig. 6) implies that the entrainment by an external zeitgeber, which is required for the presence of circalunar periodicity^25^, is lacking. At the moment this ca. 30-day cycle would therefore be more aptly described with the term circatrigintan^24^ and within that definition the reproductive cycle still shows large variation among the colonies (25- to 35-day periodicity).

It is possible that the timing of sperm release and internal fertilization in this brooding coral are more tightly controlled by the lunar cycle. Few studies have touched upon the timing of sperm production and release in this species^13,39^ and, to our knowledge, no study has been done on the possible periodicity of sperm release in this species. Hence, this possibility would require further study. If fertilization rates vary in sync with the lunar cycle but subsequent rates of larval development vary among the brooded planula larvae^13,19,42^, this developmental variation could also cause larval release at different times during the lunar cycle, which would offer an interesting explanation for the observed circatrigintan pattern of larval release.

The presence of some sort of free running endogenous clock, controlling the planulae output of shallow water *S. pistillata* in the northern Red Sea, might be a vestige from earlier times when the lunar cycle did act as zeitgeber. The intense light pollution at night in Eilat^43^, potential impacts of global changes causing shifts in reproductive seasonality^10^ or perhaps a low evolutionary need for entrainment of larval release with the lunar cycle in the Gulf of Eilat (Aqaba) could be possible reasons why the reproductive cycle of this brooding species has been decoupled from the lunar cycle. This would also explain why some of the colonies do not have a pronounced periodicity in their larval reproductive output. The lack of a strict zeitgeber for larval release and the variation in circatrigintan patterns displayed by *S. pistillata* colonies sheds new light on the discussion on coral plasticity and fecundity, and the possible implications for other brooding coral species that have previously been thought to display a tight lunar periodicity.

## Methods

*S. pistillata* planulae were collected from a coral reef in the Gulf of Eilat (Aqaba), located in front of the Interuniversity Institute for Marine Sciences (IUI) in Eilat, at a depth of 3–5 metres from 1st of April to 24th of June 2011. Planulae traps were placed at nightfall covering the coral colonies and collected the following morning with their contents, as described^30,37^. Eight large *S. pistillata* colonies (Fig. 1) were chosen haphazardly, situated no more than 20 metres apart. Initially only colonies #1–#4 were sampled in the period 1–13 April 2011. From the 14^th^ of April onwards all eight colonies were sampled with small breaks in between (4 × 1-day breaks, 1 × 2-day break, 1 × 3-day break and 2 × 6-day breaks). The traps for colonies #5–#8 experienced technical failures from 19 to 22 April, and therefore the data collected during that time were not taken into analysis.

The collected nets were retrieved from the coral colonies and immediately transferred on land to a cool box on wheels with seawater. The traps were re-suspended to minimize the time exposed to air and desiccation, and transported to a wet lab situated nearby (less than 1 minute away). Each trap was rinsed separately with seawater, and the collected planulae were flushed from each trap to a separate glass container. The planulae were counted using a stereoscope and a pipette.

Auto-correlation^44^ was applied to establish the presence of periodic patterns in larval production.

IUI data loggers collected data on site every 10 minutes, which are readily available via a website^45^. The data included water level (cm), water temperature (°C), UV irradiance (mmol m^−2^ sec^−1^) and solar irradiance (W m^−2^). Tidal range was calculated as the difference between high tide and low tide, maximum daily solar and UV irradiance were obtained from the irradiance data, and lunar period was calculated as:

$${\rm{y}}=\,\cos (2\pi \times \frac{Days\,since\,last\,full\,moon}{{\rm{\Delta }}full\,moon\,to\,full\,moon\,in\,days})$$

Cross-correlation was applied to investigate relationships between fluctuations in larval production and fluctuations in these environmental variables. Furthermore, we calculated daily solar insolation (kWh m^−2^ day^−1^), which was compared to peak reproduction timing. Images of the coral colonies were analysed using ImageJ software, and an ecological volume index was established for each colony, by approximating colonial structures to the shape of a half sphere^46^. Graphs were created in Sigmaplot 12.5 and statistical analysis were performed with SPSS 21.
